# Potential Role of NEU1 in Hepatocellular Carcinoma: A Study Based on Comprehensive Bioinformatical Analysis

**DOI:** 10.3389/fmolb.2021.651525

**Published:** 2021-08-26

**Authors:** Zhulin Wu, Li He, Lina Yang, Xuehong Fang, Lisheng Peng

**Affiliations:** ^1^ The Fourth Clinical Medical College of Guangzhou University of Chinese Medicine, Shenzhen, China; ^2^ Department of Oncology and Haematology, Shenzhen Traditional Chinese Medicine Hospital, Shenzhen, China; ^3^ Guangdong Medical University, Zhanjiang, China; ^4^ Department of Science and Education, Shenzhen Traditional Chinese Medicine Hospital, Shenzhen, China

**Keywords:** liver cancer, data mining, NEU1, biomarker, prognosis

## Abstract

**Background:** Aberrant expression of NEU1 has been identified in many malignancies. Nevertheless, the clinical significance of NEU1 in hepatocellular carcinoma (HCC) has not been fully elucidated.

**Methods:** In our study, multiple databases, including ONCOMINE, The Cancer Genome Atlas (TCGA), Gene Expression Omnibus (GEO), International Cancer Genome Consortium (ICGC), Cancer Cell Line Encyclopedia (CCLE), Human Protein Atlas (HPA), Kaplan-Meier (KM) plotter, MethSurv, Gene Expression Profiling Interactive Analysis (GEPIA), and Metascape, etc., were utilized to investigate the expression, prognostic value, and function of NEU1 in HCC.

**Results:** ONCOMINE, GEO, and TCGA analyses revealed that NEU1 was more highly expressed in HCC compared to normal tissues. Additionally, the mRNA and protein expression levels of NEU1 were increased in liver cancer cell lines and HCC tissues, respectively. Moreover, a trend toward increased NEU1 expression with advanced stage or grade was found. Furthermore, higher mRNA expression of NEU1 was found to be remarkably correlated with worse survival in HCC patients, and multivariate Cox analysis indicated that high mRNA expression of NEU1 was an independent prognostic factor for poor prognosis of HCC patients. Also, 21 methylated CpGs were found to be significantly related to HCC prognosis. Besides, functional enrichment analyses indicated that high NEU1 expression group had lower levels of B cells, CD8^+^ T cells, NK cells, and T helper cells, etc. than the low NEU1 expression group, and NEU1 may regulate a variety of tumor-related proteins and pathways, including lysosome, spliceosome, mTOR signaling pathway and so on.

**Conclusion:** High expression level of NEU1 was positively correlated with unfavorable prognosis of HCC patients, which may be related to the regulation of cancer-associated pathways and the inhibition of immune function by NEU1. Thus, NEU1 could be used as a potential prognostic biomarker and target for HCC.

## Introduction

According to recent data from GLOBOCAN, liver cancer is one of the most common malignancies with high mortality (Bray et al., 2018). Hepatocellular carcinoma (HCC) is the most common type of liver cancer, accounting for the majority (about 70–90%) of cases (Schoenberg et al., 2018). In addition, the 5-year survival rates of HCC ranges from 3 to 28%. (Li et al., 2018). Most patients with HCC are at intermediate or advanced stages when diagnosed and are not suitable for surgical treatment (Wu et al., 2020a). In 2017, regorafenib and nivolumab were approved for HCC patients, and in 2018, lenvatinib was also approved for the treatment of unresectable HCC (Jindal et al., 2019). However, due to drug resistance and side effects, the overall therapeutic effect of these drugs is unsatisfactory (Jia et al., 2020). Therefore, effective markers for the early detection, diagnosis, and prognosis of HCC are urgently needed.

A recent report has suggested that neuraminidase-1 (Neu1) could be a novel therapeutic target for cancer treatment and is expected to become an intervention for multi-stage cancer development (Haxho et al., 2016). NEU1 gene is known to be a member of human sialidases (Bonten et al., 1996), which is located on chromosome 6p21.3 and accumulates the gangliosides and glycoproteins that can lead to cell necrosis and cytotoxicity (Ren et al., 2016). Previous research reported that the expression of NEU1 was overexpressed in NSCLC patients with mutant TP53 and was related to adverse clinical outcomes (Lv et al., 2020). Moreover, targeting NEU1 can affect the proliferation, apoptosis, and epithelial-mesenchymal transition of breast cancer cells, and then changes the sialic acid level (Thulasiraman et al., 2019). Although it has been found that overexpression of NEU1 was correlated with HCC progression (Hou et al., 2016), the prognostic value and functions of NEU1 in HCC have not been intensively studied.

Based on previous findings, further analysis of NEU1 may help improve the diagnosis and prognosis of HCC. In this research, A comprehensive bioinformatical analysis was performed to assess the role of NEU1 in HCC.

## Methods

### ONCOMINE Analysis of NEU1 Expression in HCC

In the present study, ONCOMINE (www.oncomine.org) (Rhodes et al., 2007) was utilized to analyze the difference of NEU1 expression between normal and HCC tissue samples. In ONCOMINE analysis, the screening criteria were set as follows: cancer type = Hepatocellular Carcinoma; gene = NEU1; data type = mRNA; analysis type = Cancer versus Normal Analysis; threshold values: *p*-value < 1E-4, fold change > 2, gene rank = top10%. Students’*t*-test was performed to detect the difference between the normal tissue group and the HCC group. Moreover, the meta-analysis of NEU1 expression data was also performed in ONCOMINE.

### Validation of NEU1 Expression by GEO and TCGA Datasets

Publically available Gene Expression Omnibus (GEO) (Barrett et al., 2013) datasets GSE45436 (Wang et al., 2013), GSE62232 (Schulze et al., 2015), GSE76427 (Grinchuk et al., 2018), GSE101685, and GSE121248 (Wang et al., 2007) were used for NEU1 expression analysis. These gene expression data were downloaded from the bioinformatics array research tool (BART, http://igc1.salk.edu:3838/bart/) (Amaral et al., 2018). In addition, The Cancer Genome Atlas (TCGA) gene expression data were also analyzed, and the HCC dataset was obtained from TCGA data portal (https://tcga-data.nci.nih.gov/tcga/) (Weinstein et al., 2013). Data were visualized using R (version 4.0.3) packages “limma” and “beeswarm,” and Wilcox test was used to analyze statistical significance.

### Cancer Cell Line Encyclopedia and Human Protein Atlas Analyses

NEU1 expression levels in various cancer cell lines were analyzed using Cancer Cell Line Encyclopedia (CCLE) database (https://portals.broadinstitute.org/ccle) (Ghandi et al., 2019). The CCLE includes information on 1,457 human cancer cell lines and 84,434 genes and contains data visualization. Furthermore, the Human Protein Atlas (HPA) database (https://www.proteinatlas.org) was used for identifying the expression level of NEU1 protein in HCC through immunohistochemistry (IHC) staining. Also, the IHC images were downloaded from HPA. The HPA is an online tool that contains IHC test results showing the distribution and expression of proteins in various human normal tissues and tumor tissues (Asplund et al., 2012).

### Correlation Between NEU1 Expression and Clinical Factors in HCC

After completing the analyses of NEU1 protein and mRNA expression levels, correlations between NEU1 and clinicopathologic parameters were evaluated using TCGA HCC dataset. Prior to analysis, TCGA HCC patients whose clinical information (survival status, age, sex, T stage, pathologic stage, grade) was incomplete were excluded. NEU1 expression differences were analyzed by Mann-Whitney *U* test or Kruskal-Wallis test when appropriate, and data statistical analysis and result visualization were done with R software.

### Independent Prognostic Analysis of NEU1 Expression

To assess the independent prognostic value of NEU1, univariate and multivariate Cox analyses were performed for both NEU1 and clinical data using TCGA HCC dataset. For external validation, the mRNA expression and clinical information of another HCC dataset were downloaded from International Cancer Genome Consortium (ICGC) database (https://dcc.icgc.org/projects/LIRI-JP) to validate the independent prognostic value of NEU1. Clinical factors (age, sex, stage, and grade) of HCC patients in the TCGA cohort and the age, sex, and stage data of HCC patients in the ICGC cohort were extracted. By using univariate and multivariate Cox regression models, these clinical factors were analyzed in combination with the NEU1 expression. In our study, the principal outcome was overall survival (OS) defined as death from any cause, and hazard ratios (HR) with their 95% confidence intervals (95% CI) were computed based on Cox regression models. Besides, factors with *p* < 0.1 in univariate Cox regression model were chosen for multivariate analysis, and the results were visualized using R package “ggplot2.”

### Prognostic Value of mRNA Expression and DNA Methylation of NEU1

Association between NEU1 expression and prognosis was also assessed by using the online software Kaplan-Meier (KM) plotter (Menyhárt et al., 2018; Nagy et al., 2018). The KM plotter could be used to analyze the effect of more than 54,000 genes on survival in various cancers and generate KM survival curves (http://kmplot.com/analysis/). In survival analysis, patients with HCC from TCGA dataset were classified into two groups according to the optimal cutoff value of the expression of NEU1, and disease-specific survival (DSS), progression-free survival (PFS), and OS were evaluated with log-rank *p*-value and HR with 95% CI. Subgroup analyses based on pathologic stage and T stage were also performed. In KM plotter, the number of HCC patients in T4 stage group or pathologic stage 4 group was too small to be further analyzed. Additionally, the different DNA methylated sites of NEU1 were determined, and the DNA methylation-based survival analyses were performed by using the TCGA dataset in MethSurv (https://biit.cs.ut.ee/methsurv/) (Modhukur et al., 2018) which is an online tool for methylation visualization.

### Immune Regulating Roles of NEU1

Based on the TCGA dataset, single-sample gene set enrichment analysis (ssGSEA) was utilized to compare the enrichment scores of immune cells and immune-related pathways between high and low expression subgroups based on the median expression level of NEU1 by using “GSVA,” “GSEABase” packages R software (Hänzelmann et al., 2013). Besides, tumor mutation burden (TMB) which is computed as the total mutation incidences per million base pair is a reliable biomarker for predicting sensitivity to immune checkpoint inhibitors (Liu et al., 2020), and we used Spearman’s correlation analysis to analyze the association between NEU1 and TMB score. Data processing, statistical analysis, and data visualization were done with an online bioinformatics analysis tool from China (Assistant for Clinical Bioinformatics, https://www.aclbi.com/).

### Functional Enrichment Analysis by Metascape Database

The method of functional analysis referred to previous research (Wu et al., 2020b). Before enrichment analysis, similar genes of NEU1 were acquired using the Gene Expression Profiling Interactive Analysis (GEPIA, http://gepia.cancer-pku.cn/) (Tang et al., 2017). Functions of NUE1 and its similar genes were analyzed using Metascape database (http://metascape.org) which is a powerful online database for gene functional annotation analysis and is updated monthly (Zhou et al., 2019). In Metascape, Gene Ontology (GO) analysis can determine the functions of NEU1 and its similar genes on the basis of three categories, containing molecular functions, cellular components, and biological processes, while Kyoto Encyclopedia of Genes and Genomes (KEGG) analysis can identify the signaling pathways associated with NEU1 and its similar genes. Besides, Protein-protein interaction (PPI) and independent functional enrichment analyses of Molecular Complex Detection (MCODE) components were conducted using Metascape. In this paper, *p*-value < 0.05 denotes differences that are statistically significant if not stated otherwise.

## Results

### NEU1 is Distinctively Overexpressed in HCC

**FIGURE 1:**
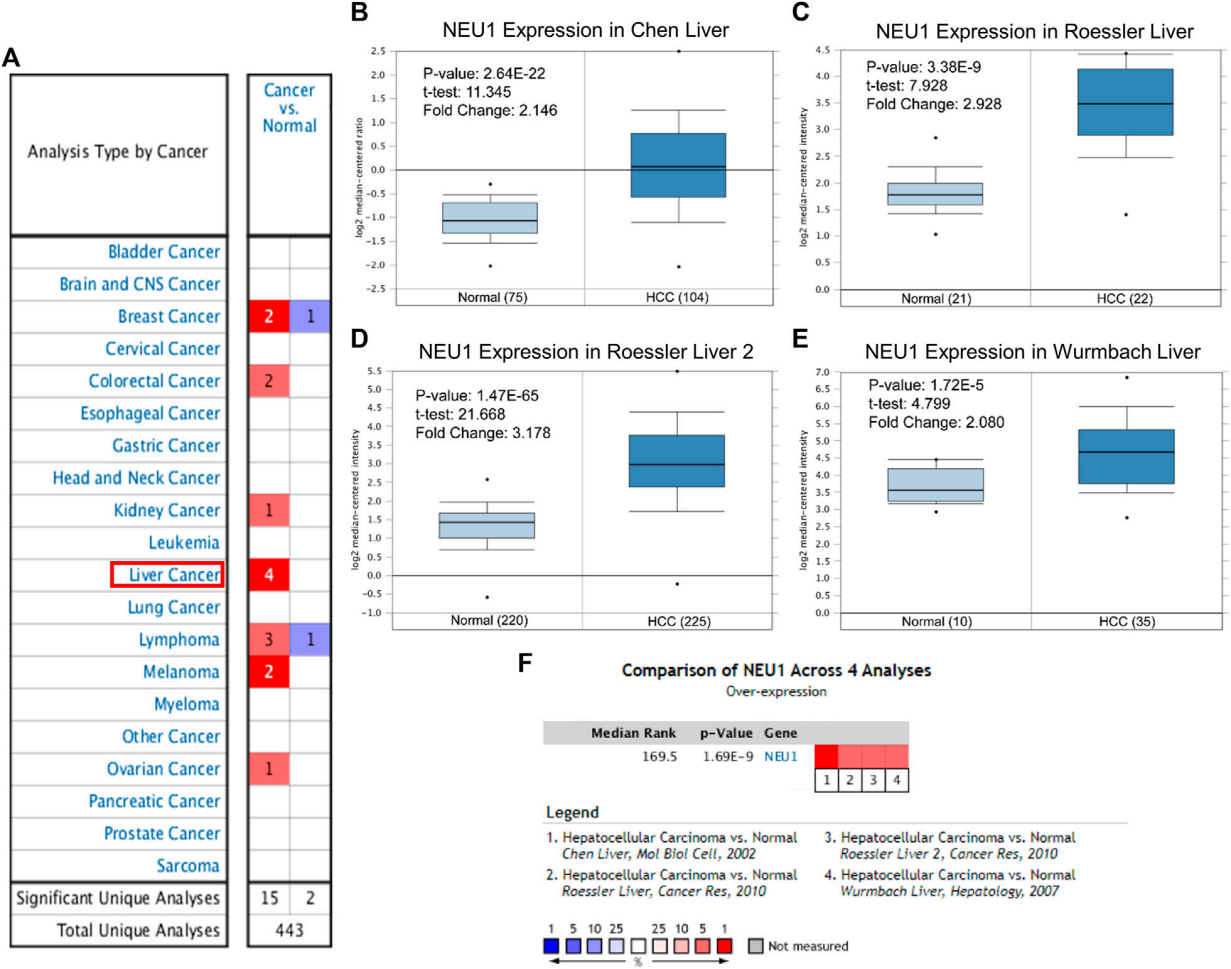
The mRNA expression levels of NEU1 in multiple cancers and HCC based on ONCOMINE database **(A)** The mRNA expression pattern of NEU1 in various cancer types. Red color and blue color respectively indicate high and low expression. The number in each box represents the number of datasets that fulfill the threshold **(B–E)** The box-plots derived from four different datasets comparing NEU1 expression in normal **(left)** and HCC tissues **(right)**. HCC tissues showed higher NEU1 expression compared with normal liver tissues **(F)** A meta-analysis of NEU1 expression from four ONCOMINE datasets (*p* = 1.69E-9).

A total of 443 research results were analyzed in ONCOMINE, of which 17 research (high NEU1 expression in 15 research, and low NEU1 expression in two research) fulfilled the screening criteria mentioned above (Figure 1A). The result showed that there were four datasets with high expression of NEU1 in liver cancer, and these datasets comprised a total of 712 samples (386 HCC samples and 326 normal samples) (Chen et al., 2002; Wurmbach et al., 2007; Roessler et al., 2010). Furthermore, all the four datasets in ONCOMINE demonstrated that NEU1 mRNA expression was remarkably elevated in HCC than that in normal samples, and the data of sample size, fold change, and *p*-value corresponding to the four studies are also summarized in Figures 1B–E. Besides, a meta‐analysis of the four datasets from ONCOMINE was performed, and the result revealed that NEU1 was expressed highly in HCC compared with the normal groups (*p*-value = 1.69E-9, Figure 1F).

**FIGURE 2:**
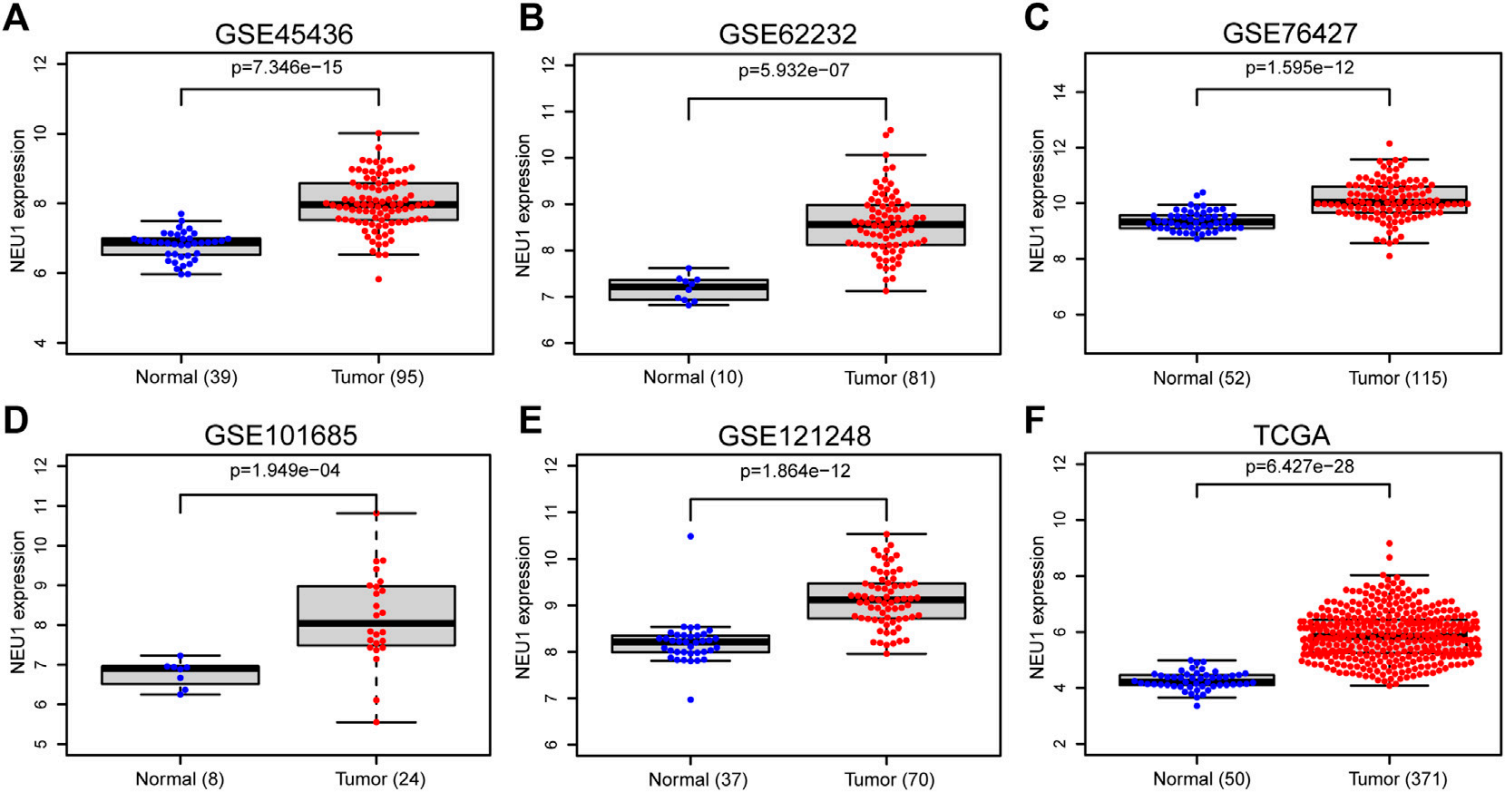
Expression of NEU1 in HCC and normal tissues (TCGA and GEO datasets) **(A)** GSE45436 dataset; **(B)** GSE62232 dataset; **(C)** GSE76427 dataset; **(D)** GSE101685 dataset; **(E)** GSE121248 dataset; **(F)** TCGA dataset.

To verify NEU1 mRNA expression, five GEO datasets and the TCGA dataset of HCC were also analyzed, and the information about those GEO datasets is listed in Supplementary Table S1. As illustrated in Figures 2A–F, NEU1 mRNA was remarkably overexpressed in GSE45436, GSE62232, GSE76427, GSE101685, GSE121248, and TCGA datasets.

### NEU1 Expression in HCC Cell Lines and NEU1 Protein Expression in HCC

**FIGURE 3:**
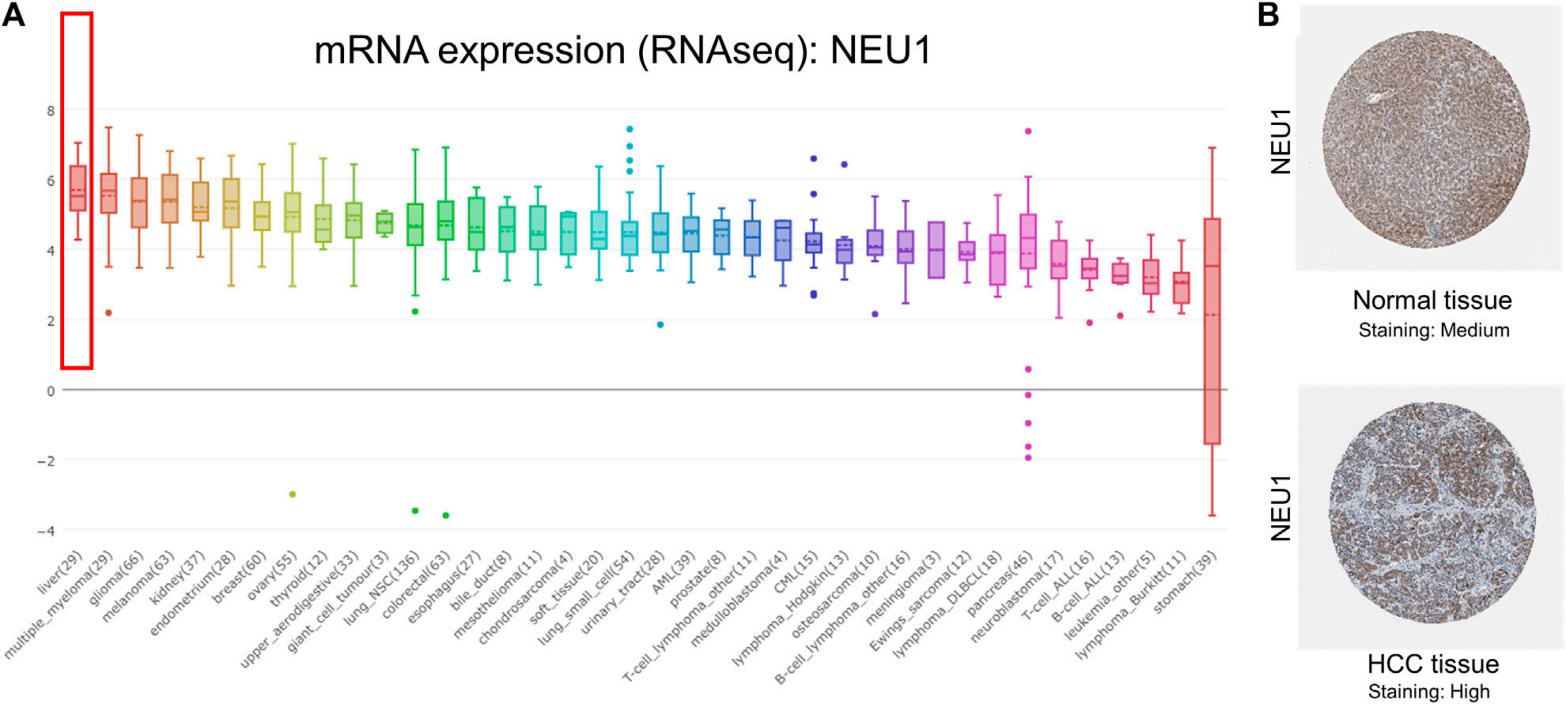
Results from the Cancer Cell Line Encyclopedia (CCLE) and Human Protein Atlas (HPA) analyses of NEU1 **(A)** NEU1 was highly expressed in liver cancer cell lines (top 1) **(B)** Representative images of the immunohistochemical staining of NEU1 in HCC tissue and normal liver tissue (Antibody: HPA015634). Medium protein expression of NEU1 was found in normal liver tissue, while high protein expression was observed in HCC tissue.

The result of CCLE analysis revealed that mRNA expression level of NEU1 in cell lines of liver cancer listed the first highest among all tumor types (Figure 3A). By using HPA platform, representative images of the immunohistochemical staining of NEU1 in HCC tissue and normal liver tissue were obtained (Antibody HPA015634 in HPA database), and medium protein expression of NEU1 was determined in normal liver tissue, while high protein expression was identified in HCC tissue (Figure 3B).

### Correlation Between Clinical Features and the Expression of NEU1

**FIGURE 4:**
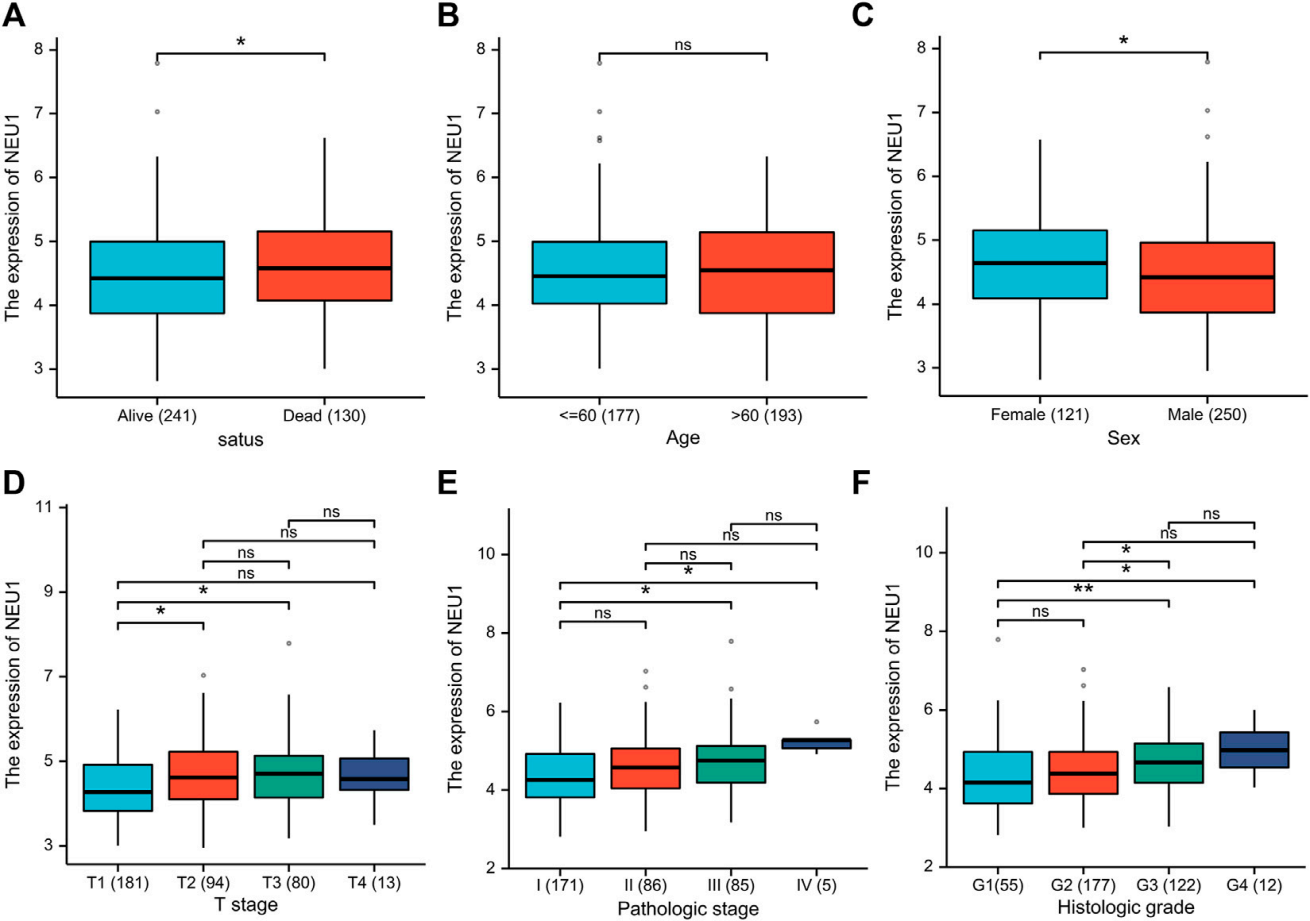
The relationship between the NEU1 expression and clinicopathologic parameters (TCGA dataset) **(A)** Relative expression of NEU1 in alive status or dead status **(B)** Relative expression of NEU1 in younger patients (≤ 60) or elder patients (> 60) **(C)** Relative expression of NEU1 in female or male **(D)** Relative expression of NEU1 in T stage 1, 2, 3, or 4 **(E)** Relative expression of NEU1 in pathologic stage I, II, III, or IV **(F)** Relative expression of NEU1 grade 1, 2, 3, or 4. ns represents *p* ≥ 0.05; * represents *p* < 0.05, and ** represents *p* < 0.01.

In order to identify correlations of NEU1 expression with clinical features (survival status, age, sex, T stage, pathologic stage, histologic grade), the clinical data in the TCGA database were analyzed (Figures 4A–F). In terms of OS events, NEU1 was overexpressed in dead samples compared to alive samples (Figure 4A). Differences in NEU1 expression between females and males were also significant (Figure 4B). As presented in Figures 4A,D–F trend toward increased NEU1 expression with advanced pathologic stage, T stage, or histologic grade was observed. Besides, there is no significant difference in NEU1 expression according to age (Figure 4B).

### Association Between NEU1 and Prognosis in HCC Patients

**FIGURE 5:**
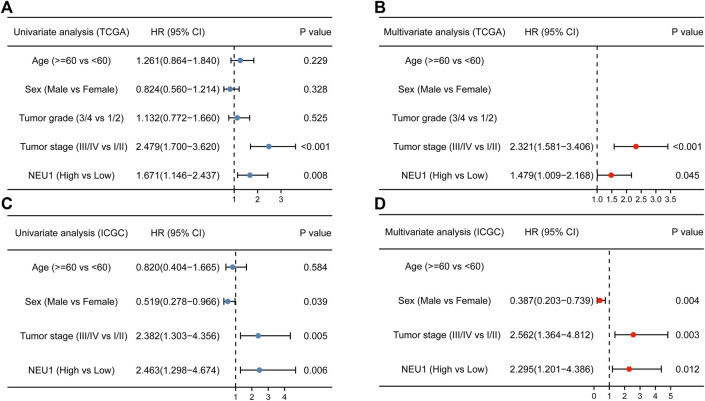
Results of univariate and multivariate Cox analyses of clinical factors and NEU1 expression **(A)** Forest plot of univariate analysis in TCGA cohort **(B)** Forest plot of multivariate analysis in TCGA cohort **(C)** Forest plot of univariate analysis in ICGC cohort **(D)** Forest plot of multivariate analysis in ICGC cohort.

After the relationship between NEU1 expression and OS event was confirmed, the correlation between NEU1 and prognosis was further explored. In univariate Cox analysis of the TCGA cohort, we found that advanced stages and high mRNA expression of NEU1 were correlated with shorter OS of patients with HCC (Figure 5A). Furthermore, multivariate Cox analysis indicated that high expression of NEU1 was independently correlated with significantly wore OS of patients with HCC (HR = 1.479, 95% CI = 1.009–2.168, *p* = 0.045, Figure 5B). Moreover, the univariate and multivariate Cox analyses of the ICGC cohort also revealed that high mRNA expression of NEU1 was related to worse OS (Figures 5C,D), and the expression of NEU1 was an independent prognostic factor for predicting OS in multivariate Cox regression analysis (HR = 2.295, 95% CI = 1.201–4.386, *p* = 0.012).

**FIGURE 6:**
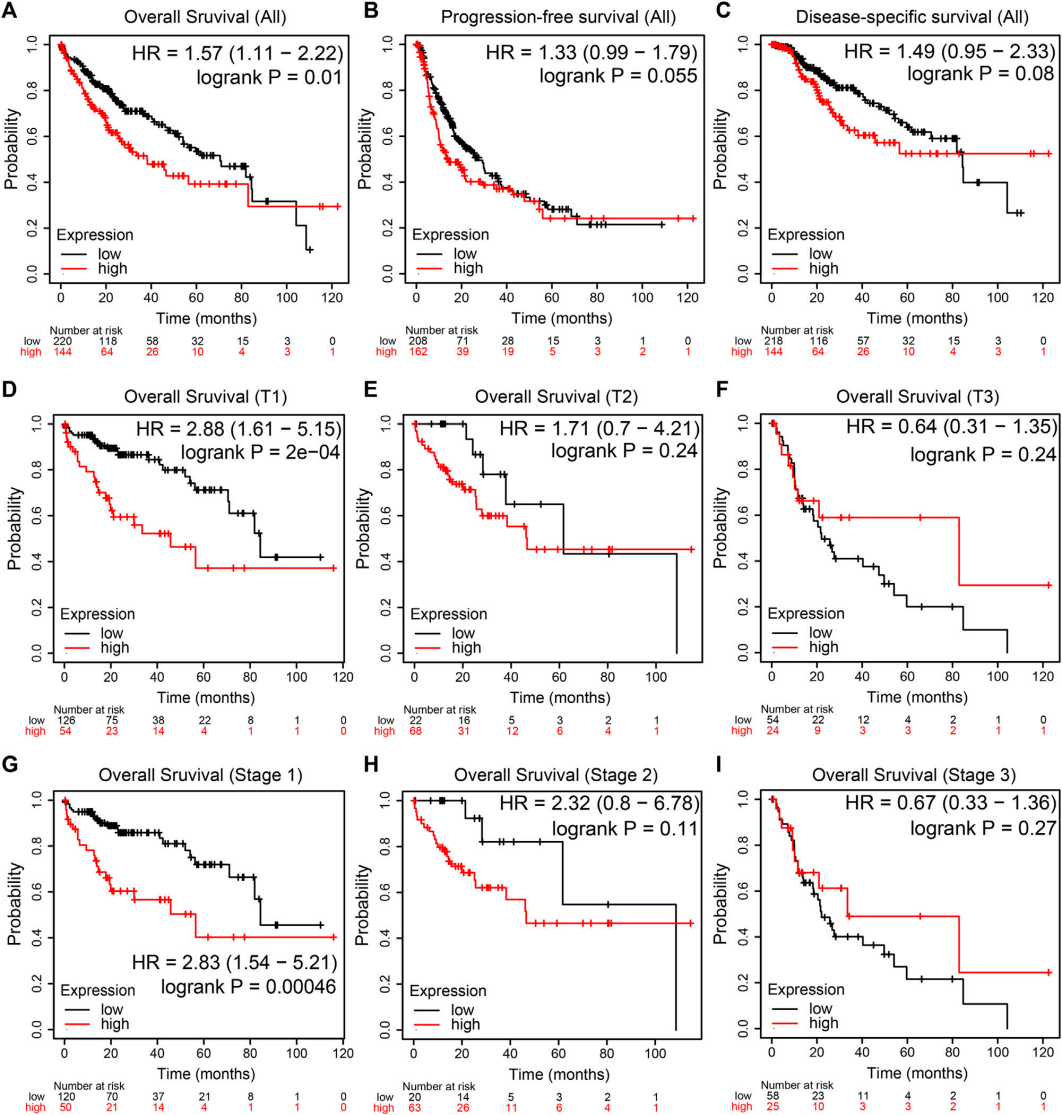
The prognostic value of NEU1 in patients with HCC (TCGA cohort) **(A–I)** The Kaplan-Meier survival curves comparing HCC patients with low (black) and high (red) NEU1 expression were plotted **(A,D,G)** High expression level of NEU1 was significantly correlated with shorter OS in all HCC, T1, and stage 1 patients (*p*-value < 0.05) **(B–C)** The expression of NEU1 was not related to PFS and DSS in all HCC patients (*p*-value > 0.05) **(E–F, H–I)** NEU1 expression showed no correlation with OS in other T stages and pathologic stages.

**FIGURE 7:**
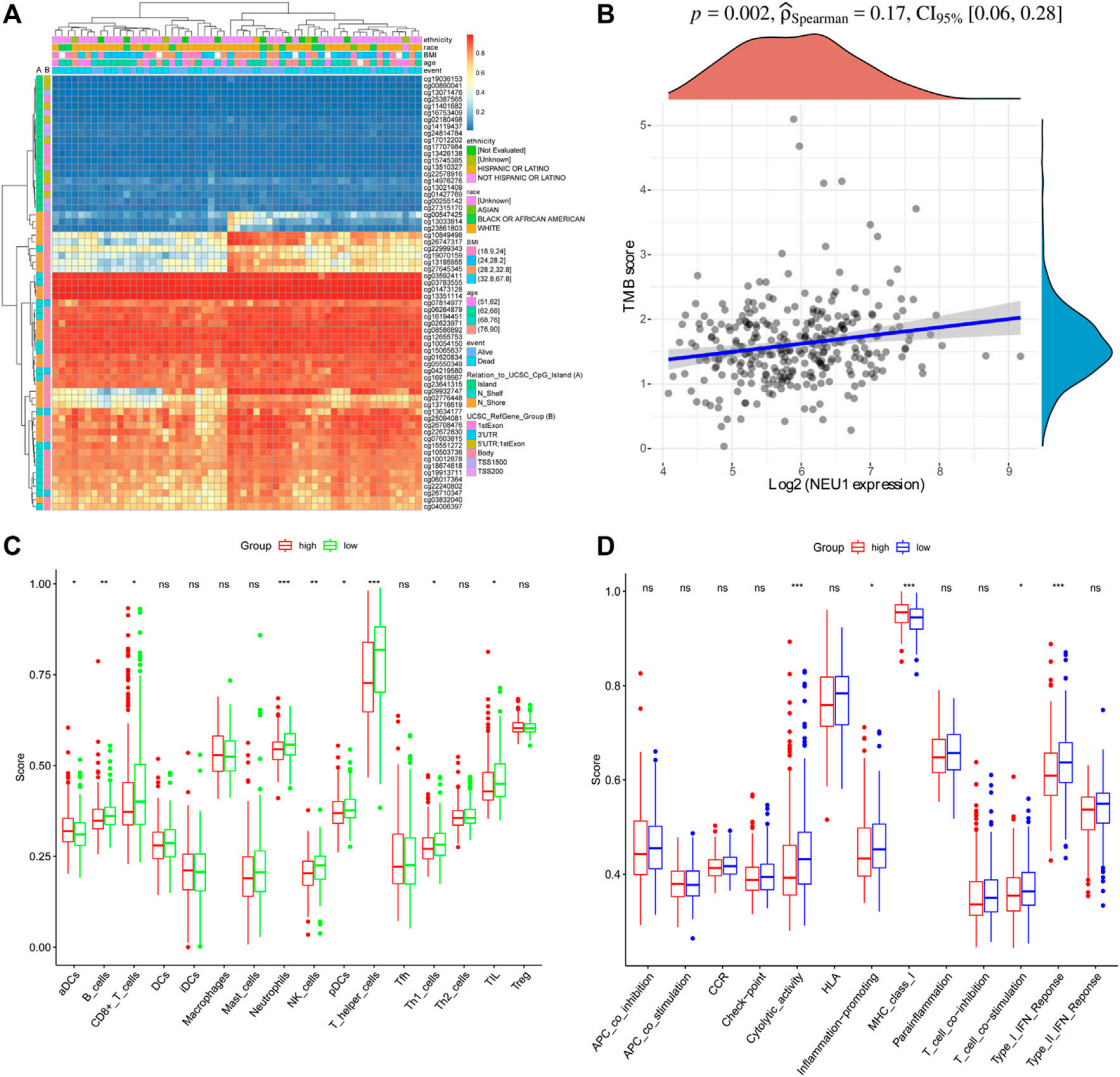
DNA methylation of NEU1 and immune regulating roles of NEU1 (TCGA dataset) **(A)** The DNA methylation clustered expression of NEU1. Blue to red color represents low to high expression, and different color side boxes denoted ethnicity, race, age, Body mass index (BMI), event, UCSC RefGene Group, and Relation to UCSC CpG island **(B)** Correlation analysis of NEU1 expression and TMB score. The density curve on the right indicates the distribution trend of the TMB score; the upper density curve indicates the distribution trend of the gene **(C)** The scores of 16 types of immune cells between high and low NEU1 expression groups **(D)** The scores of 13 types immune-associated pathways between high and low NEU1 expression groups. ns represents *p* ≥ 0.05; *p < 0.05; **p < 0.01; ***p < 0.001.

The prognostic value of NEU1 in HCC was also evaluated using KM plotter, and the results are shown in Figures 6A–I. The KM plot showed that high expression of NEU1 was related to the shorter OS in all HCC patients (HR = 1.57, *p* = 0.01; Figure 6A). However, the association between NUE1 expression and PFS in all HCC patients did not attain statistical significance (Figure 6B), and a similar result was obtained for DSS (Figure 6C). Moreover, subgroup analysis demonstrated that high NEU1 expression was correlated with worse OS time in both T1 and stage 1 HCC patients [(HR = 2.88, p = 2e-04) and (HR = 2.83, *p* = 0.00046), respectively] (Figures 6D,G). No other subgroup analyses were statistically significant. Also, the DNA methylation level of NEU1 and prognostic values of CpGs were analyzed using MethSurv. As illustrated in Figure 7A, cg13351114 showed the highest DNA methylation. Moreover, 21 CpGs of NEU1 were related to the prognosis of patients with HCC (Table 1).

**TABLE 1:** The prognostic values of CpGs in NEU1 (MethSurv database).

Gene-CpG	HR	*p*-value	Gene-CpG	HR	*p*-value
Island-TSS200-cg00255142	1.1	0.64	N_Shore-Body-cg13195955	0.696	0.046^a^
Island-TSS200-cg13510327	0.96	0.82	N_Shore-Body-cg13351114	0.643	0.024^a^
Island-TSS200-cg14119437	1.335	0.13	N_Shore-Body-cg13716619	0.861	0.41
Island-TSS200-cg16753409	1.03	0.86	N_Shore-Body-cg19070159	0.673	0.048^a^
Island-5′UTR;1stExon-cg01427769	1.408	0.051	N_Shore-Body-cg22672830	0.547	0.002^a^
Island-5′UTR;1stExon-cg02180498	0.53	1.119	N_Shore-Body-cg23641315	1.469	0.062
Island-5′UTR;1stExon-cg11401682	1.181	0.41	N_Shore-Body-cg23861803	0.638	0.014^a^
Island-5′UTR;1stExon-cg14976276	1.122	0.57	N_Shore-Body-cg25094081	0.788	0.25
Island-5′UTR;1stExon-cg17012202	1.209	0.34	N_Shore-Body-cg26708476	0.644	0.031^a^
Island-5′UTR;1stExon-cg19036153	1.877	0.003^a^	N_Shore-Body-cg26747317	0.586	0.006^a^
Island-5′UTR;1stExon-cg22578916	1.643	0.02^a^	N_Shore-Body-cg27645345	0.775	0.2
Island-1stExon-cg13021409	1.584	0.024^a^	N_Shelf-Body-cg03592411	1.388	0.12
Island-1stExon-cg13426138	1.584	0.36	N_Shelf-Body-cg03783555	1.344	0.093
Island-1stExon-cg17707984	0.95	0.77	N_Shelf-Body-cg04006397	1.11	0.63
Island-1stExon-cg25387565	1.75	0.008^a^	N_Shelf-Body-cg06017364	0.887	0.49
Island-TSS1500-cg13071476	0.965	0.85	N_Shelf-Body-cg06264879	0.775	0.21
Island-TSS1500-cg24814784	1.271	0.21	N_Shelf-Body-cg10012878	1.423	0.069
Island-TSS1500-cg27315170	0.842	0.33	N_Shelf-Body-cg10054150	0.694	0.059
N_Shore-Body-cg00547425	0.863	0.47	N_Shelf-Body-cg10503736	0.854	0.43
N_Shore-Body-cg01620834	1.302	0.13	N_Shelf-Body-cg15065837	0.696	0.04^a^
N_Shore-Body-cg02623971	1.673	0.015^a^	N_Shelf-Body-cg16194451	1.085	0.64
N_Shore-Body-cg02776448	0.711	0.051	N_Shelf-Body-cg16918667	0.605	0.013^a^
N_Shore-Body-cg03832040	0.606	0.005^a^	N_Shelf-Body-cg18674618	1.36	0.082
N_Shore-Body-cg05550349	1.237	0.23	N_Shelf-Body-cg19913711	0.818	0.27
N_Shore-Body-cg07603815	1.243	0.24	N_Shelf-Body-cg22240802	0.784	0.23
N_Shore-Body-cg08586892	1.714	0.003^a^	N_Shelf-Body-cg22999343	1.241	0.31
N_Shore-Body-cg09932747	0.639	0.018^a^	N_Shelf-3′UTR-cg04219580	1.119	0.59
N_Shore-Body-cg10849498	0.656	0.019^a^	N_Shelf-3′UTR-cg13634177	0.689	0.035^a^
N_Shore-Body-cg12655753	1.473	0.031^a^	N_Shelf-3′UTR-cg15551272	0.43	0.854
N_Shore-Body-cg13033814	0.757	0.16	N_Shelf-3′UTR-cg26710347	0.681	0.029^a^

a represents *p* < 0.05.

### Analysis Result of Immune Regulating Roles of NEU1

As shown in Figure 7B, the result of Spearman’s correlation analysis indicated the existence of a positive correlation between NEU1 expression and TMB score (*p* = 0.002). To further investigate the relationship between NEU1 expression and immune status, we quantified the enrichment scores of different immune cell subsets, associated functions, or pathways with ssGSEA. In 16 types of immune cells, the scores of aDCs, B cells, CD8^+^ T cells, Neutrophils, pDCs, NK cells, T helper cells, Th1 cells, and TIL were significantly different between high and low NEU1 expression groups (*p*-value <0.05, Figure 7C). In 13 types of immune-associated pathways, the score of cytolytic activity, inflammation promoting, MHC class I, T cell co-stimulation, and Type I IFN response were remarkably different between high and low NEU1 expression groups (*p* < 0.05, Figure 7D).

### Functional Enrichment Analyses of NEU1 in HCC

Before conducting enrichment analyses, the top 100 similar genes of NEU1 were obtained from GEPIA (Supplementary Table S2). The functions of NEU1 and its similar genes were predicted using Metascape, and the thresholds of the Min Overlap, *p*-value, and Min Enrichment in the database were set to 3, 0.05, and 3, respectively.

**FIGURE 8:**
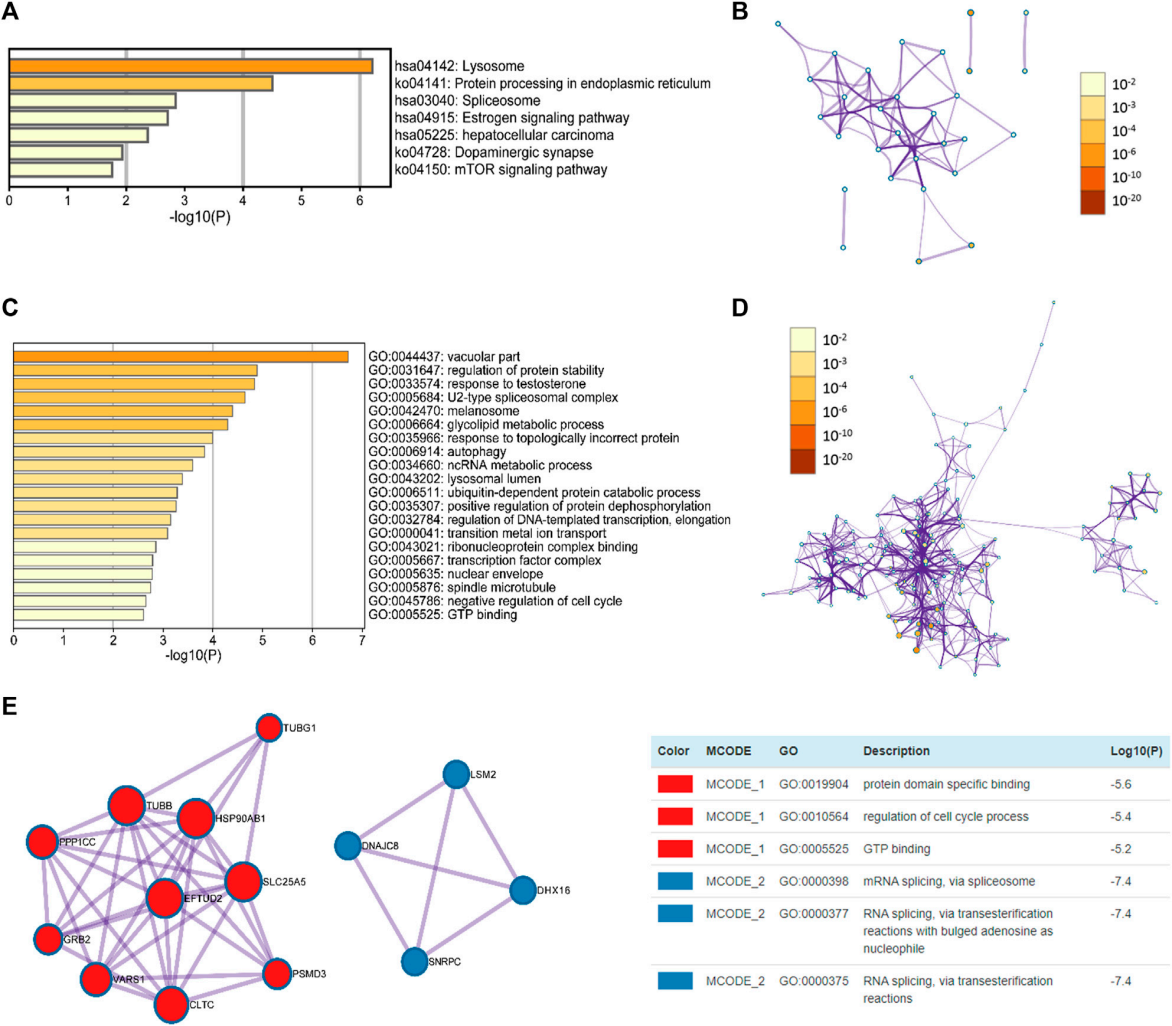
Enrichment analysis of NEU1 and similar genes in HCC patients (GEPIA and Metascape) **(A)** The heatmap of GO terms **(B)** The network of GO terms **(C)** The heatmap of KEGG terms **(D)** The network of KEGG terms **(E)** Two key MCODE components form the protein-protein interaction (PPI) network and independent functional enrichment analysis of MCODE components.

The top seven KEGG pathways with a significant *p* < 0.05 are shown in Figures 8A,B, and these seven items included lysosome, protein processing in endoplasmic reticulum, spliceosome, estrogen signaling pathway, hepatocellular carcinoma, dopaminergic synapse, and mTOR signaling pathway. As shown in Figures 8C,D, the enriched GO terms were categorized into three groups: biological process group (11 GO terms), molecular function group (7 GO terms), and cellular component group (2 GO terms). These genes were mainly enriched in vacuolar part, U2-type spliceosomal complex, regulation of protein stability, response to testosterone, ribonucleoprotein complex binding, and GTP binding, etc.

Furthermore, to further examine the association between these genes and HCC, a Metascape PPI enrichment analysis was conducted, and the results of PPI network and MCODE components are presented in Figure 8E. The two most important MCODE components were obtained from the PPI analysis. Following pathway and process enrichment analyses of each MCODE component independently, we found that biological function was mainly correlated with protein domain specific binding, regulation of cell cycle process, and GTP binding, etc.

## Discussion

HCC is one of the most refractory malignant tumors, and HCC patients often have a poor prognosis because of late diagnosis. Thus, novel potential diagnostic and prognostic markers are urgently needed. Previous research reported that sialidase NEU1 is expressed at different levels in normal tissues and is strongly deregulated in different cancer, which showed the potential application of NEU1 in tumor diagnosis and therapy (Forcella et al., 2018). However, there have been scant studies on the correlation between NEU1 expression and human HCC, and the role of NEU1 in HCC still requires further mining.

Our study first investigated the expression of NEU1 in HCC with the ONCOMINE, GEO, and TCGA datasets, and the findings showed that NEU1 is highly expressed in HCC tissues in comparison with normal tissues. Moreover, the enhanced expression of NEU1 was confirmed in liver cancer cell lines, and we also found that NEU1 protein expression was upregulated in HCC tissues compared with normal tissues (HPA). Previous studies from two different groups have implied that NEU1 is upregulated in hepatitis B-associated HCC (Wang et al., 2009; Amaddeo et al., 2015), which supported our findings. Furthermore, we observed that the NEU1 expression was significantly related to the T stage, pathologic stages, and grades, and a trend toward increased NEU1 expression with advanced cancer stages or grades. Recently, it has been found that NEU1 promoted the proliferation and migration of HCC by activating intracellular signaling pathways (Kong et al., 2020), and siRNA-mediated silencing of NEU1 was related to the decrease of proliferation and migration of HCC cells (Hou et al., 2016). Also, it was observed that there was a discrepancy between male and female, and the underlying mechanism need further investigation. Besides, NEU1 was upregulated in dead samples compared to alive samples, so we further analyze the prognostic role of NEU1 to evaluate its clinical utility.

In survival analysis, the KM curve revealed a notably shorter OS for all HCC patients with high NEU1 levels than those with low NEU1 levels. A trend toward poor PFS or DSS was seen in patients with high NEU1 expression, but this did not reach statistical significance. In stratified analyses, we found that NUE1 increased predicted worse OS in T1 stage and pathologic stage 1 subgroups, and the expression of NEU1 showed no statistical significance for the OS of pathologic stage 2/3 or T2/3 HCC patients. More importantly, not only univariate but also multivariate analyses in both TCGA and ICGC cohorts showed that NEU1 was an independent prognostic factor for patients with HCC. It has been reported that NEU1 could regulate epithelial mesenchymal transformation which is thought to be involved in the invasion and metastasis of cancer, leading to poor prognosis (Wang et al., 2015). Accumulating research on genome-wide DNA methylation strongly supported the importance of differences in methylation of CpG sites between HCC tissues and paracancerous tissues (Wang et al., 2020). By using the MethSurv database, a total of 60 methylated CpG sites of NEU1 were identified, and over one-third of CpG sites were presented with prognostic value in HCC patients. These findings revealed that NEU1 may have clinical utility as a prognostic marker for HCC.

Although the correlation was not high, it is found that the expression of NEU1 was positively associated with the TMB score. Previous studies have indicated TMB is significantly correlated with the prognosis of HCC patients (Shrestha et al., 2018) and is a predictive biomarker related to immunotherapy responses in cancers (Xie et al., 2020). Thus, future research on TMB should also pay attention to the role of NEU1. Moreover, the ssGSEA analysis showed that high NEU1 expression group had lower levels of B cells, CD8^+^ T cells, neutrophils, NK cells, pDCs, T helper cells, Th1 cells, and TIL than the low NEU1 expression group. The levels of antitumor infiltrating immune cells were low, indicating that the immune function of HCC patients with high NEU1 expression is generally impaired. Immune dysfunction was observed in patients with malignant tumors, which is one of the reasons for tumor progression (Najima et al., 2016). Specifically, tumor-infiltrating CD8^+^ T cell responses are thought to be associated with a good prognosis of HCC patients (Hofmann et al., 2021), and a significant positive correlation has been reported between the total number of NK cells and a favorable prognosis of HCC patients (Sun et al., 2017). Based on these findings, the poor prognosis of HCC patients with high NEU1 expression may be caused by decreased levels of overall antitumor immunity.

To further reveal the function of NUE1 and its similar genes, functional enrichment analysis was performed and pathways involving lysosome, protein processing in endoplasmic reticulum, spliceosome, estrogen signaling pathway, hepatocellular carcinoma, dopaminergic synapse, and mTOR signaling pathway were found in the KEGG analysis. It is reported that hyperactivated lysosomes caused cancer and induced metastasis or tumor relapse (Radisavljevic, 2019), and tumor microenvironment could cause the redistribution of lysosomes toward the cell periphery, which enhanced invasion and metastasis by exocytosis of lysosomal hydrolases (Ballabio and Bonifacino, 2020). An experimental study demonstrated that NEU1 siRNA can significantly suppress proliferation, apoptosis, and invasion of tumor cells by targeting lysosomal membrane proteins (CLN3 and CLN5) (Ren et al., 2016). One recent study indicated that genomic mutation, such as somatic changes in the genes encoding components of the spliceosome, often occurred in human tumors (Palangat et al., 2019), and a meta-analysis has shown that many genes of spliceosome pathway were up-regulated in HCC (Xu et al., 2015). Additionally, targeting the spliceosome could reduce the proliferation of cancer cells (van Alphen et al., 2009). Recently, it has been found that the existence of rapid estrogen signaling in HCC could promote tumor growth (You et al., 2018). Hyperactivation of mTOR could promote the cell growth and metabolism that lead to tumor progression (Tian et al., 2019). The PI3K/Akt/mTOR is an important signal pathway in HCC carcinogenesis and plays a core role in promoting tumor cell proliferation (Xing et al., 2020). Moreover, hyperactivated lysosome causes permanent activation of AKT, which then controls lysosome and directly regulates cancer development and metastasis (Radisavljevic, 2019). Besides, the GO and PPI analyses demonstrated that NEU1 and its similar genes were involved in various biological processes and molecular functions, and some of them (e.g., autophagy, ncRNA metabolic process, and regulation of cell cycle process) were correlated with tumorigenesis. These results revealed that NEU1 could promote proliferation, migration, and invasion of HCC by regulating various tumor-associated proteins and pathways. However, there were some deficiencies in the present study. The findings of survival analyses were based on databases, and more validations are warranted to verify our findings. Also, our research lacked experimental verification.

In general, NEU1 was significantly overexpressed in HCC tissues compared with normal liver tissues, suggesting that NEU1 may be used as a potential biomarker for HCC. Moreover, high expression of NEU1 could be a predictor of poor prognosis in HCC, especially in early-stage patients, and NEU1 was also an independent risk factor for the prognosis of HCC. This may be related to the regulation of cancer-associated pathways and the inhibition of immune function by NEU1. Additionally, multiple DNA methylated sites of NEU1 could provide prognosis assessment for HCC patients.

## Data Availability

The original contributions presented in the study are included in the article/Supplementary Material, further inquiries can be directed to the corresponding author.
