# Supplementary material for: Potential Role of NEU1 in Hepatocellular Carcinoma: A Study Based on Comprehensive Bioinformatical Analysis
Source: Front Mol Biosci. 2021 Aug 26;8:651525. doi: 10.3389/fmolb.2021.651525 (PMC8427823; doi:10.3389/fmolb.2021.651525)
Supplement: Supplementary file 1 [file DataSheet1.docx]

Supplementary Material

# Supplementary Tables

**Supplementary Table 1.** Information of included GEO datasets.

| **Dataset ID** | **Sample size** | **Platforms** |
| --- | --- | --- |
| GSE45436 | normal: 39; HCC: 95 | GPL570 [HG-U133_Plus_2] Affymetrix Human Genome U133 Plus 2.0 Array |
| GSE62232 | normal: 10; HCC: 81 | GPL570 [HG-U133_Plus_2] Affymetrix Human Genome U133 Plus 2.0 Array |
| GSE76427 | normal: 52; HCC: 115 | GPL10558 Illumina HumanHT-12 V4.0 expression beadchip |
| GSE101685 | normal: 8; HCC: 24 | GPL570 [HG-U133_Plus_2] Affymetrix Human Genome U133 Plus 2.0 Array |
| GSE121248 | normal: 37; HCC: 70 | GPL570 [HG-U133_Plus_2] Affymetrix Human Genome U133 Plus 2.0 Array |

**Supplementary Table 2.** 100 similar genes of NEU1 (GEPIA).

| Similar genes extracted from GEPIA |
| --- |
| NELFE,CSNK2B,AGPAT1,RNF187,ABCF1,PPP1R11,RPP21,MEA1,BAG6,EHMT2,ATP6AP1,NOL7,C6orf47,SLC35B2,CDKAL1,SNRPC,NFKBIL1,GLMP,LSM2,NABP2,TUBB,CUTA,ATF6B,GNL1,GNPDA1,UTP18,RNF8,CLTC,CCHCR1,CTSA,RRP36,FKBPL,SLC39A7,MIEN1,RPN2,FLOT1,ZNHIT3,PIGT,RABIF,HTATSF1,GPANK1,ZBTB9,CDK4,MTCH1,TBC1D7,RNF5,NKAP,PPP1CC,TCF19,RALY,PSPH,SLC39A1,VARS,TMEM115,MAD2L1BP,FAM220A,PIGU,PPP2R5D,GRB2,GRN,PRIM2,CYB561D2,TAF11,MFSD5,DNAJC8,ABHD16A,CLIC1,MRPS23,KLHDC3,EFTUD2,SKA1,SMARCE1,SLC26A11,PSMD3,SLC25A39,SAE1,ZNRD1,SLC25A5,DHX16,SLC41A3,DDIT3,RRAGC,DTNBP1,ABT1,AVL9,TUBG1,TOMM6,PDZD11,PHB,PPIAP22,PIP4K2C,RPN1,GBA,HSP90AB1,TMEM101,KPNA2,WRNIP1,GNS,STMN1,DDAH2 |
